# Neuroendocrine differentiation and neuroendocrine morphology as two different patterns in large-cell bronchial carcinomas: outcome after complete resection

**DOI:** 10.1186/1477-7819-4-61

**Published:** 2006-09-05

**Authors:** Wolfgang Jungraithmayr, Gian Kayser, Bernward Passlick, Stephan Eggeling

**Affiliations:** 1 Department of Thoracic Surgery, University Hospital Freiburg, Hugstetter Str. 55, 79106 Freiburg, Germany; 2 Department of Pathology, University Hospital Freiburg, Breisacher Str. 115a, 79106 Freiburg, Germany

## Abstract

**Background:**

In 1999, large-cell neuroendocrine carcinoma of the lung was introduced by the World Health Organization (WHO) as a new tumor entity in the group of non-small cell, epithelial tumors, a differentiated classification of neuroendocrine tumors of the lung not existing until this time. Scientific knowledge on prognosis and therapy of these tumors, especially between those with neuroendocrine morphology only and those showing additional expression of neuroendocrine markers, is fragmentary. In this analysis, we studied the clinical behavior and the prognosis of these two rare tumor entities.

**Patients and Methods:**

The analysis comprises 12 patients of a total of 2053, who underwent thoracotomy for non small-cell lung carcinoma between 1997 and 2005 in the Department of Thoracic Surgery at the University Hospital of Freiburg. Clinical data, pathological examinations as well as complete follow-up were reviewed from large-cell carcinoma with neuroendocrine morphology only (n=4) and from large-cell carcinoma expressing neuroendocrine markers (n=8).

**Results:**

The median survival of patients with neuroendocrine morphology was 30 months (11–96 months). In the patient group showing the expression of neuroendocrine markers, the median survival time was 20 months (2–26 months). Tumor recurrences occurred in the group with neuroendocrine morphology, without exception, in the form of distant metastases and in the group with neuroendocrine markers as intrapulmonary metastases.

**Conclusion:**

Large-cell neuroendocrine carcinomas of the lung show aggressive behavior with a poor prognosis. Expression of neuroendocrine markers markedly reduce tumor-free interval as well as survival and might influence the site of metastases.

## Background

Morphologically and clinically-pathologically, neuroendocrine tumors of the lung form a heterogeneous group of pulmonary carcinomas. According to the classification by the World Health Organization (WHO) of 1981, the carcinoid and the small-cell carcinoma were categorized together as neuroendocrine tumors. In 1999, at the recommendation of the WHO as well as the International Association for Study of Lung Cancer (IASLC) a distinction was made in the carcinoids into typical and atypical carcinoid tumors, the large-cell neuroendocrine carcinoma of the lung being included as a new tumor entity in its own right [1]. From a clinical-prognostic point of view, this tumor group can be categorized as being between the atypical carcinoid and the small-cell carcinoma, tending more towards the small-cell carcinoma, however [2-4]. Morphologically, in comparison to these parallels are to be found, above all, with respect to the incidence of mitosis and necrosis. The differentiating feature between the two tumors in conformity with the names they have been given indicates the cell size as well as the ratio of the cell nucleus to the cell plasma. The also newly classified so-called "combined large-cell neuroendocrine carcinoma" describes all non-small-cell bronchial carcinomas, which exhibit neuroendocrine morphological characteristics to only a limited degree, overall, however, keeping their non-small-cell basic structure. In the past, for the sake of simplicity, this group of tumors was categorized together in the group of so-called mixed tumors. Decisive for the correct classification as a large-cell carcinoma with neuroendocrine differentiation (Large Cell Neuro-Endocrine Carcinoma or LCNEC) is electron microscopic characterization or the detection of at least one immunohistochemical marker [5]. On the other hand, tumors characterized as large-cell neuroendocrine tumors merely on the basis of morphology, i.e., using light microscopy, can be described as large-cell carcinomas with neuroendocrine morphology (LCCNM) [6]. The basis for differentiating between these two groups of tumors has been provided by Zacharias and Iyoda [6,7]. Taking this distinction into account, we studied the prognostic and therapeutic significance of these large-cell neuroendocrine carcinomas.

## Patients and methods

The analysis comprises 12 patients with LCCNM or LCNEC of a total of 2053 patients, who underwent thoracotomy for non-small-cell carcinomas between 1997 and 2005; this represents a percentage of 0.6%. Preoperatively, computed tomography of the thorax, as well as diagnostic flexible bronchoscopy were performed in all cases. All patients underwent curative surgery with systematic lymphadenectomy, the tumor stage being classified according to the UICC classification system.

Histopathologically, hematoxylin-eosin staining as well as an immunohistochemical examination to differentiate between LCCNM and LCNEC were conducted on a routine basis. To do this, antibodies for the markers synaptophysin, neurone-specific enolase (NSE), CD56 and chromogranin A were used on formalin-fixed as well as paraffin-embedded tissue sections.

The analysis took into account age and sex, type of operation, postoperative stage as well as the long-term course of the patients. Due to the small number of patients, no formal statistical analysis of the data was carried out.

## Results

### Pathology

**Figure 1:**
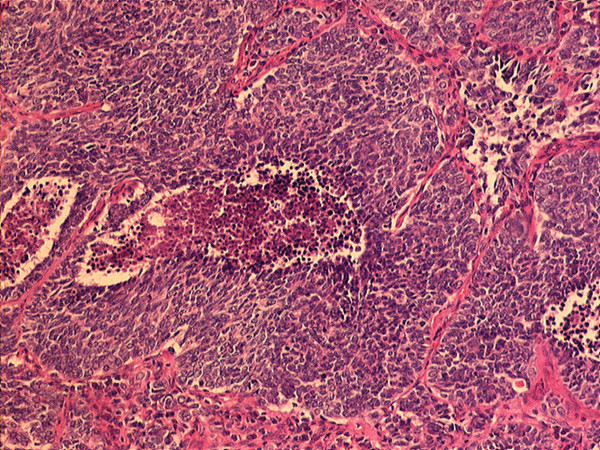
Large-cell neuroendocrine carcinoma with formation of typical spherical cell clusters, in which tumor cells are arranged partly trabecularly, partly in the form of a rosette. (Magnification: 100×, Stain: Hematoxylin-Eosin).

**Figure 2:**
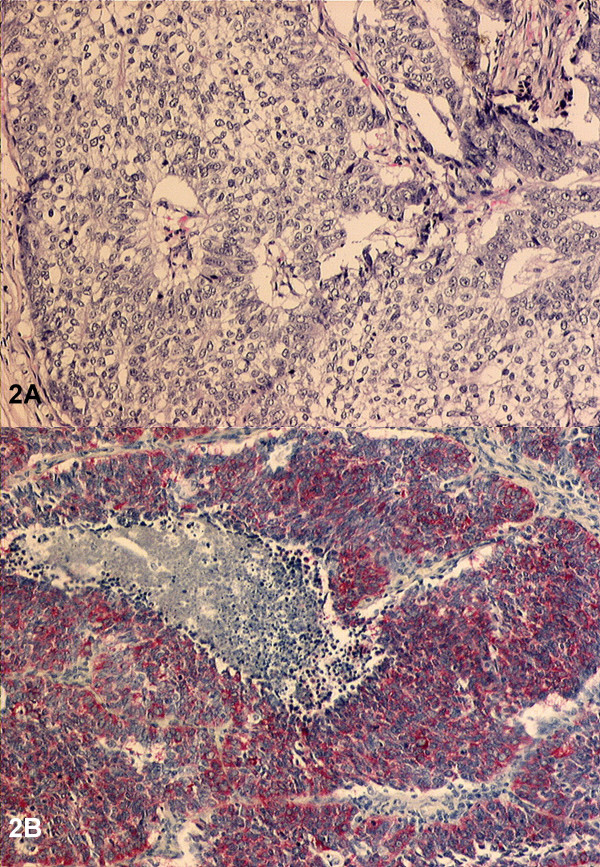
Immunohistochemical staining for neurone-specific enolase (NSE) of a large-cell carcinoma with neuroendocrine morphology (A) and a large-cell neuroendocrine carcinoma (B). (Magnification: 100×, Stain: NSE).

Using routine staining, an adenoid or squamous epithelial differentiation in the group of large-cell carcinomas was excluded. Only those carcinomas were included in the study which showed the histomorphologically typical growth patterns of neuroendocrine tumours such as spherical cell clusters and rosettiform arrangement of the tumor cells (Figure 1). Neuroendocrine differentiation could then accordingly be demonstated or excluded immunohistochemically (e.g. for NSE: Figure 2). Of 12 patients, in 8 patients at least one neuroendocrine marker could be determined, these thus being classified as LCNEC (Table 1).

**Table 1:** Incidence and distribution of neuroendocrine markers in LCNEC.

	**LCCNM**	**LCNEC**
*Neuroendocrine Marker*	-	+
Synaptophysin	-	71 %
NSE	-	57 %
CD56	-	43 %
Chromogranin A	-	14 %

NSE = Neuron-Specific Enolase.

### Clinical data

All patient characteristics are described in detail in Table 2. Twelve patients underwent surgery during the period from May 1997 to February 2005 in the Department of Thoracic Surgery at the Freiburg University Clinic (♀ = 1, ♂ = 11). The mean age was 65 years (41–80). The exact diagnosis of a large-cell carcinoma with either neuroendocrine morphology or neuroendocrine expression could not be made preoperatively using bronchoscopy.

**Table 2:** Characteristics of patients with large-cell carcinoma and neuroendocrine marker expression as well as with large-cell carcinoma and neuroendocrine morphology only.

**Patient**	**M/F**	**Age**	**Stage**	**Resection**	**Follow-up (Months)**	**Postoperative Diagnosis**	**Status**
**1**	M	41	II B	LL l	60	LCCNM	† intrapulmonary recurrence *
**2**	M	57	I B	LL r	96	LCCNM	Disease-free
**3**	M	76	I B	LL l	30	LCCNM	† intrapulmonary recurrence *
**4**	M	74	III A	UL r	11	LCCNM	† intrapulmonary recurrence *

**5**	M	61	I A	S 4/5 l	18	LCNEC	Disease-free
**6**	M	66	I B	Pneumonectomy l	26	LCNEC	† distant metastases
**7**	M	75	I A	UL l	24	LCNEC	Disease-free
**8**	M	67	I B	LL l	20	LCNEC	† brain metastases
**9**	M	70	II B	Pneumonectomy r	12	LCNEC	† bone metastases
**10**	F	58	IV	UL l	20	LCNEC	† bone metastases
**11**	M	80	I A	S 8 r	19	LCNEC	Disease-free
**12**	M	57	III B	Bifurcation	2	LCNEC	Disease-free

M = male; F = female; LL = lower lobe; UL = upper lobe; l = left; r = right; S = segment; † = deceased; * = intrapulmonary metastases.

In 4 cases a right-sided, in 7 cases a left-sided operation was carried out, and in one case a bifurcation resection in a centrally located tumor. The tumor stage in the group with LCCNM was I B in two patients, II B in one patient and III A in one patient. In the group of patients with LCNEC, the tumor stage was I A in three cases, I B in two cases, and II B, III B and IV in one case each. In the group of patients with LCCNM, a lobectomy was performed in four cases. In the group of patients with LCNEC, a lobectomy was performed three times, a pneumonectomy twice due to the central location of the tumor, a segment resection twice because of inadequate pulmonary function as well as a bifurcation resection in a centrally located carina carcinoma. A systematic lymphadenectomy was performed in all patients. Adjuvant chemotherapy was given to patients in tumor stage II a, and more advanced stages.

The median follow-up was 20 months (2–96 months). The median survival time in the group of patients with LCCNM was 30 months (11–96 months), one patient still being alive. In the group of patients with LCNEC, the median survival time was 20 months (2–26 months), 4 patients still being alive. The 1-year survival rate (1-YSR) in the group with LCCNM was 40% and in the group with LCNEC 50%. The median tumor-free survival time in the group of patients with LCCNM was 41 months (7–96 months), in the group of patients with LCNEC 12 months (6–24 months). At the time of evaluation one patient in the group of patients with LCCNM was still alive without recurrence in a good general state of health, 3 patients died as a result of intrapulmonary metastases. In the group of patients with an LCNEC, 4 patients are still alive without recurrence in a good general state of health and 4 patients died as a result of distant metastases. Tumor recurrences occurred in the group with LCCNM, without exception, as intrapulmonary metastases and in the group with LCNEC in the form of non-pulmonary distant metastases exclusively.

## Discussion

In the WHO version of 1981, the carcinoid and the small-cell carcinoma only were categorized under the term neuroendocrine tumors. In 1985, Carter called for a more precise differentiation of neuroendocrine tumors of the lung [8]; in an elaborate immunohistochemical analysis of non-small-cell carcinomas, Linnoila identified several neuroendocrine markers, especially in large-cell tumors [9]. In a study based on this, Travis introduced the term large-cell neuroendocrine carcinoma (LCNEC) as a variant of the large-cell carcinoma and suggested this tumor be considered a new tumor entity in addition to the carcinoid as well as the small-cell carcinoma. This was characterized by light-microscopically determined phenotypically large, polygonal cells, a high incidence of mitoses and necroses, as well as the immunohistochemical or electron microscopic determination of neuroendocrine markers [5]. Due to the absence of a consensus as well as the call for reproducibility and clinical significance [10], in 1999 the WHO along with the IASLC 1999 included the large-cell neuroendocrine bronchial carcinoma as a variant of the large-cell carcinoma into the classification system [1]. Subsequently, on the basis of clearly defined criteria, an increasing number of clinical series were published giving consideration to this new type of tumor [11-14].

Based on our series of 12 patients, we studied the significance of neuroendocrine expression by considering a further difference between large-cell bronchial carcinomas with neuroendocrine morphology only (LCCNM) as well as large-cell bronchial carcinomas showing the expression neuroendocrine markers (LCNEC). These tumor groups differ only in the expression of neuroendocrine markers, the morphology determined using a light microscope, on the other hand, being identical in both tumors. Both groups show growth patterns that are generally typical for neuroendocrine tumors, independent of their site of origin. Thus, tumor cell rosettes and clearly delimited spherical cell clusters are found on a regular basis.

This subtyping is not made in the criteria formulated in 1999 by the WHO and IASLC; using a careful division into these two types of tumor, Zacharias and Iyoda, however, show that this stratification is of importance with respect to prognosis and therapy [6,7]. In both the uni-as well as in the multivariate analysis, Iyoda was able to demonstrate a worse overall as well as tumor-free survival time in patients showing the expression of neuroendocrine markers [7]. Travis demonstrated a relationship between increased CEA in the serum and shorter survival times [5]. These observations concur with our results on the tumor-free intervals, which were almost four times shorter in patients with LCNEC. In contrast, Harada *et al*, showed an improved survival time in such tumors in which more than 2 neuroendocrine markers could be identified immunohistochemically [15]. The 1-year survival rate in the LCCNM group was 40%, in the LCNEC group 50%. In the largest series published so far on large-cell neuroendocrine bronchial carcinomas by Takei [11], the 5-year survival (YS) in stage I was 67%, in stage IV 0%. In our series, only 2 patients survived 5 years in a tumor stage I B and II B, all the other patients died within the first 2 postoperative years. Jiang reports on a stage-dependent 1-YS of 58.8%, which is comparable to our LCNEC 1-YS. The poor prognosis of this tumor becomes clearer when one considers the 1-YS of 86.2% in patients with NSCLC undergoing surgery [16], although the overall poor median survival cannot solely attributed to the neuroendocrine nature of these tumors but also to the advanced tumor stages of the patients in this series.

The clinically aggressive behavior of the LCNEC was also demonstrated by Iyoda by a significantly enhanced expression of Bcl-2. He could observe also a shorter tumor-free survival times [17].

The exact preoperative diagnosis using a bronchoscopic or transthoracic biopsy proved unsuccessful in all our cases. The success of a definitive diagnosis of LCNEC preoperatively is also judged to be extremely difficult by other authors as well [13]. Wiatrowska and Kakinuma report on reliable data based on cytological material [18,19]. Whether a cytological diagnosis is adequate to determine a therapy concept remains uncertain.

In the group of patients with LCNEC, we could demonstrate that one tumor recurrence occurred in the form of a distant metastasis exclusively, whereas in the group of patients with an LCCNM there was a regular occurrence of intrapulmonary metastasis. This observation is not to be found in any of the clinical series published to date; it is possible that neuroendocrine markers have an influence on the metastatic process as well as the site of metastasis. Due to the small number of patients involved, this trend is not subject to any statistical analysis.

With respect to its severity, LCCNM and LCNEC are classified as being between the atypical carcinoid and the small-cell carcinoma, whereby some authors ascribe these types of tumors a greater similarity to the small-cell carcinoma. There are features in common regarding the high incidence of mitosis and necrosis as well as several phenotypic characteristics; small-cell carcinomas can also express a similar spectrum of endocrine markers. Until present, LCNEC were treated in a similar way to NSCLC, incorporating adjuvant chemotherapy in a fixed treatment regimen, however, remaining up until now not being taken into account. In light of the small number of cases in the reported series to date, the advantage of such a form of chemotherapy must be evaluated as still being uncertain. Whereas in a clinical study in patients with non-small cell bronchial carcinoma and neuroendocrine differentiation, an improved survival time as a result of adjuvant chemotherapy was not reported [2], Iyoda was able to demonstrate an improvement of survival time of about 20% in stage I with adjuvant chemotherapy [20]. Until now there have been no studies on this providing a randomized comparison of the group undergoing surgery with and without adjuvant chemotherapy.

Another adjuvant therapeutic approach already described before was taken up again by Caretta in a study on 44 neuroendocrine carcinomas, in which he points out the importance of the octreotide scan [21]. Filosso was able to demonstrate a significant increase in survival time with adjuvant octreotide therapy in 55% of scintigraphically positive patients [22].

## Conclusion

The tumor groups LCCNM and LCNEC show aggressive behavior with a poor prognosis. In spite of the small number of our series, we observe tendencies: tumors expressing neuroendocrine markers have a considerably shorter recurrence-free survival time in comparison to the group of patients showing neuroendocrine morphology only. Another remarkable feature is the way of relapse. Tumor recurrence in the LCNEC group occurred exclusively in the form of distant metastases and in the group of patients with LCCNM, on the other hand, in the form of an intrapulmonary metastasis.

## Competing interests

There is no personal, political or financial competing interest in the material, information or techniques described in the paper.

## Authors' contributions

**GK **was involved with the histomorphological analysis of the specimens and in writing the study. **BP and SE **critically reviewed the paper and were involved in the preparation of the final manuscript. **WJ **prepared the manuscript.

All authors have read and approved the final version of the manuscript
